# Efficiency of polymyxin B treatment against nosocomial infection: a systematic review and meta-analysis

**DOI:** 10.3389/fmed.2024.1400757

**Published:** 2024-05-28

**Authors:** Liyuan Peng, Zhongheng Zhang, Xueyan Qi, Yanjun Zhong, Tongwen Sun, Lvlin Chen, Junchen Zhu, Xiangui Lv, Penglin Ma

**Affiliations:** ^1^Department of Critical Care Medicine, Affiliated Hospital of Chengdu University, Chengdu, Sichuan, China; ^2^Department of Emergency Medicine, Sir Run Run Shaw Hospital, Zhejiang University School of Medicine, Hangzhou, Zhejiang, China; ^3^Department of Emergency and Critical Care Medicine, Henan Engineering Research Center for Critical Care Medicine, Henan Key Laboratory of Critical Care Medicine, The First Affiliated Hospital of Zhengzhou University, Zhengzhou, Henan, China; ^4^Critical Care Medicine, The Second Xiangya Hospital, Changsha, Hunan, China; ^5^Department of Critical Care Medicine, Guiqian International General Hospital, Guiyang, Guizhou, China

**Keywords:** nosocomial infections, Polymyxin B, colistin, tigecycline, ceftazidime-avibactam, meta-analysis

## Abstract

**Background:**

Some cohort studies have explored the effects and safety of polymyxin B (PMB) in comparison to other antibiotics for the treatment of nosocomial infections, yielding inconsistent results. This systematic review aims to explore the effectiveness and safety of PMB and compared it with other antibiotics.

**Methods:**

A systematic literature search was conducted in PubMed, Embase, the Cochrane Library, and Web of Science, searching specific terms to identify quantitative cohort studies or RCTs that compared the effects of PMB with other antibiotics in terms of their efficacy and safety. The Newcastle–Ottawa Scale (NOS) was conducted to evaluate the risk of bias of observational studies. Odds ratios with 95% confidence intervals were used for outcome assessment. We evaluated heterogeneity using the *I*^2^ test.

**Results:**

A total of 22 observational trials were included in the analysis. The PMB group had a higher mortality rate compared to the control group (odds ratio: 1.84, 95% CI: 1.36–2.50, *p*<0.00001, *I*^2^ = 73%). while, the ceftazidime-avibactam group demonstrated a distinct advantage with lower mortality rates, despite still exhibiting high heterogeneity (odds ratio 2.73, 95% confidence interval 1.59–4.69; *p* = 0.0003; *I*^2^ = 53%). Additionally, the PMB group had a lower nephrotoxicity rate compared to the colistin group but exhibited high heterogeneity in the results (odds ratio 0.58, 95% CI 0.36–0.93; *p* = 0.02; *I*^2^ = 73%).

**Conclusion:**

In patients with nosocomial infections, PMB is not superior to other antibiotics in terms of mortality, specifically when compared to ceftazidime-avibactam. However, PMB demonstrated an advantage in terms of nephrotoxicity compared to colistin.

## Background

In the past decade, nosocomial infections caused by multidrug-resistant (MDR) bacteria have emerged as a significant cause of mortality and morbidity worldwide, particularly among critically ill patients (1–8). Notably, limitation in antibiotics options against MDR bacterial infections became an extreme challenge in nosocomial settings in recent decades. Estimates suggest that in the United States alone between 5 and 10% of hospitalized patients admitted to acute-care hospitals may acquire nosocomial infections (3, 4).

*Acinetobacter baumannii*, *Pseudomonas aeruginosa*, and Enterobacteriaceae are the most common MDR bacteria causing fatal nosocomial infections, especially the strains producing extended-spectrum *β*-lactamases (ESBLs), New Delhi Metallo-beta-lactamase (NDM) and Carbapenemases as well. With a growing threat of MDR bacteria, however, there have been only a few new drugs developed in recent decades, such as tigecycline, ceftazidime-avibactam and eravacycline. As instead, some old antibiotics were reassessed for their potential effectiveness against MDR Gram-negative bacteria. Of them, colistin and polymyxin B (PMB) have gained a great of attention in the last decade (9, 10). Significantly, they were reintroduced as salvage therapy for infections caused by MDR Gram-negative bacteria that did not respond to other treatments (11). Meanwhile, a controversy remains in physician’s options for their clinical uses owing to the adverse events, especially with regard to the nephrotoxicity and the neurotoxicity. For instance, PMB demonstrated a lower renal toxicity than the comparable dose of colistin in several trials (12–16). Additionally, pharmacologically experimental data demonstrated that PMB achieved effective plasma concentrations more rapidly than colistin while administrating intravenously (17, 18). Moreover, previous researches suggested that PMB showed a better efficacy in treatment of nosocomial infections caused by MDR Gram-negative bacteria including *Acinetobacter baumannii*, *Pseudomonas aeruginosa*, and CRO (12, 19). On the other hand, a few cohort studies showed that Colistin exhibited a lower mortality rate compared to PMB (12). In this systematic review and meta-analysis, therefore, we sought to study the effectiveness and safety of PMB in treatment of nosocomial infection caused by MDR bacteria comparing with colistin and other antibiotics as well.

## Method

We conducted our systematic review and meta-analysis by using predefined protocol according to the Preferred Reporting Items for Systematic Reviews and Meta-analysis (PRISMA) reporting guideline (20). The review protocol was registered in PROSPERO (CRD42023446418).

### Data sources and search strategy

A systematic literature search was conducted in PubMed, Embase, the Cochrane Library, and Web of Science from the beginning to August 1, 2023, to include clinical trials in patients of nosocomial infections associated with polymyxin B (PMB) and other antibiotics. The search only included English publications. The search terms were “Polymyxin B,” “Drug Resistance, Multiple, Bacterial,” “Aerosporin,” “mortality,” “Hospital-Acquired Pneumonia.” In addition, the reference lists of relevant studies, systematic reviews, and meta-analysis were manually examined to identify any additional publications that could contribute to our analysis. For studies that did not report complete data on our dichotomous or continuous outcomes but were eligible for inclusion in the meta-analysis, we made efforts to contact the original investigators via email in order to acquire Supplementary Data. The search strategy was presented on Supplementary Table S1 (including PubMed database, Embase, Cochrane library and Web of science).

### Study selection

Two of us (LP and XL) screened the titles and abstracts of all initially selected articles independently in accordance with the eligibility criteria. Then, the above two reviewers independently assessed full text of the selected articles from first step. Disagreements were resolved by reaching a consensus or seeking help from a third author (PM). We considered overall mortality as the primary outcome of our meta-analysis. Secondary outcomes were nephrotoxicity and microbiological eradiation.

### Inclusion criteria

We considered studies to be following criteria:

1. Enrolled were adults (≥18 years old) with nosocomial infections.
2. Treated by PMB compared with other antibiotics.
3. Outcomes (mortality, nephrotoxicity or microbiological eradiation) were available.
4. Randomized controlled trials or cohort studies.

### Data extraction and study quality assessment

Data extraction was conducted by 2 reviewers independently (YH and XL) using a standardized form and discussed with another reviewer (PM). And the following date were extracted: the study characteristics (setting, sample size), participant characteristics (sex, age, duration of medication), specification of interventions (types of pathogenic bacteria, the control group).

### Quality assessment

Two reviewers (LP and ZZ) independently conducted risk-of-bias assessments using the Newcastle-Ottawa Scale (NOS) to evaluate the observational studies included in the meta-analysis. The NOS tool assesses the risk of bias across three domains: selection, comparability, and outcome. The NOS criteria yield a quality score ranging from 0 to 9, with higher scores indicating better study quality (21, 22). Disagreements between the two investigators were resolved through discussions involving the third investigator (LC).

### Subgroup analysis

We performed subgroup analysis based on the risk of bias in the trials to optimize heterogeneity and obtain more reliable results. We also performed subgroup analysis to compare the effectiveness of PMB with different antibiotics, based on the type of antibiotic used in the control group.

### Heterogeneity

Heterogeneity was assessed visually using forest plots and quantified with I^2^ statistics, categorized as low (0–25%), moderate (26–50%), and high (>50%). If substantial heterogeneity was present and an adequate number of publications were available (n = 10), we aimed to investigate potential sources of heterogeneity through prespecified subgroup analysis outlined in the study protocol, such as restricting the analysis to high-quality studies. Additionally, a *post hoc* sensitivity analysis was conducted to assess heterogeneity after excluding studies that demonstrated a statistically significant benefit from placebo treatment.

### Publication bias

We intended to assess publication bias by utilizing funnel plots for outcomes with data available from 10 or more studies.

### Statistical analysis

All statistical analysis were performed with the use of RevMan software (version 5.3.3; The Cochrane Collaboration). The odds ratios with associated 95% confidence intervals were used to assess outcomes. Heterogeneity was assessed by the I^2^ test. Random effects models are used for all analysis. We considered *p* values <0.05 statistically significant. Comparison adjusted funnel plots are used to evaluate the possibility of study.

## Results

### Search results

**Figure 1:**
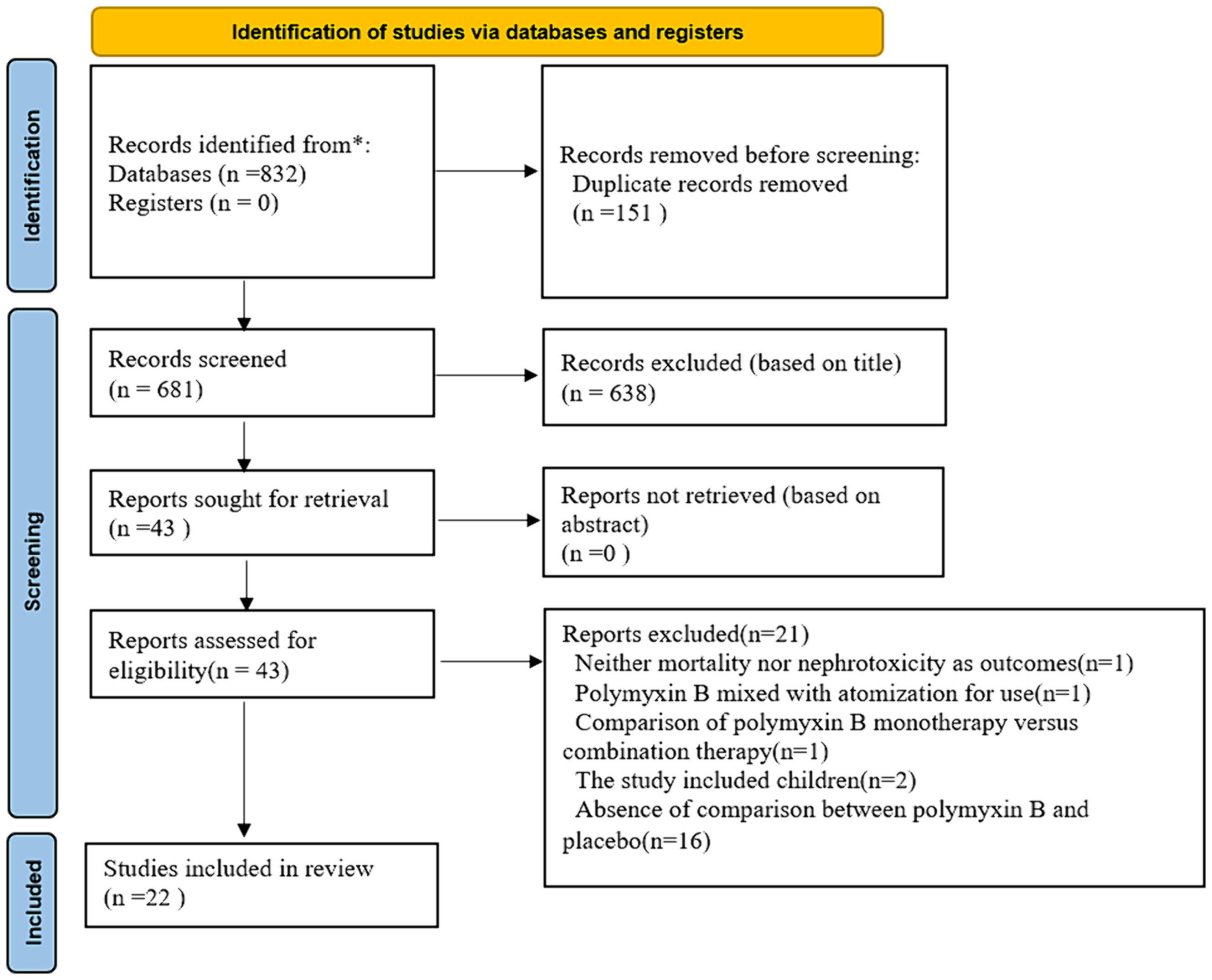
Flow chart.

We identified 832 citations and finally 22 studies met the inclusion criteria (Figure 1). The searcher strategies were shown in Supplementary Table S1. Nineteen studies reported data on the mortality, while 16 studies described nephrotoxicity.

### Study characteristics

The study characteristics are displayed in Table 1. Colistin was reported as the control group antibiotic in 9 studies, tigecycline was reported in 7 studies, and ceftazidime-avibactam was reported in 7 studies. The pathogen species, the control group and the dosage and duration of antibiotic use among all studies were shown in Table 1.

**Table 1:** Characteristics of selected clinical trials.

Author	Year	Country	Sample size	Pathogens	Study Design	Inclusion period	Infection site	Medications of control group	Duration and Dosage	Outcomes	NOS
Maura S. Oliveira (23)	2009	Brazil	41/41	CRAB	mROS	1996–2004	All site (except urinary tract)	Colistin	Daily dose of colistin was 6 MU (1–9 MU) and 1 MU (0.4–1.5) for PMB;12 days for colistin and 11 days for PMB (0–50 days)	30-day mortality, nephrotoxicity	9
Carlos H. Kvitko (24)	2011	Brazil	45/88	PA	sROS	2004–2009	All site	Others	Daily dose of PMB was 141 ± 54 mg and this drug was more frequently prescribed every 12 h	in-hospital mortality, nephrotoxicity	7
Maria Helena Rigatto (25)	2013	Brazil	45/22	PA, AB	sPOS	2009–2010	Respiratory	Others	Daily dose of PMB was 150 mg, administered every 12 h	30-day mortality, incidence of superinfection, eradication of the bacteria	9
Kady Phe (12)	2014	USA	104/121	PA, AB, KP, E.coli	mROS	2006–2011	All site	Colistin	Daily dose of PMB was 103.9 ± 40.1 mg, while the dose of colistin was 275.2 ± 106.8 mg	hospital mortality, nephrotoxicity	7
Felipe F. Tuon (13)	2014	Brazil	96/36	PA, AB, KP	sROS	2009–2011	All site	Colistin	The median (IQR) average daily dose of PMB was 2 MIU (1–2 MIU) and Colistin was 9 MIU (9–13.5 MIU)	hospital mortality, nephrotoxicity	6
Maria Helena Rigatto (14)	2016	Brazil	410/81	AB, KP, PA, E.spp., E.coli	mPOS	2013–2014	All site	Colistin	Daily dose of PMB was 150 (IQR,140 to 187) mg, administered every 12 h; Daily dose of colistin was 300 (IQR,253 to 300) mg, administered every 12 h or 8 h	30-day mortality, nephrotoxicity	8
Ryan L. Crass (26)	2017	USA	78/336	MDR-GNB	sROS	NA	All site	Colistin	NA	30-day mortality, nephrotoxicity	8
Jie Fang (27)	2021	China	78/37	CRKP	mROS	2018–2020	All site	Ceftazidime/Avibactam	PMB was administered at a dose of 1.25–1.5 mg/kg every 12 h; Ceftazidime/Avibactam 2.5 g was administered every 8 h	28-day mortality, microbiological clearance	9
Juan Chen (28)	2021	China	85/51	CRPA	sROS	2018–2020	All site	Ceftazidime/Avibactam	The duration for PMB was 9 (5–12) days, while for colistin was 10 (6–17) days	4-day mortality, 30-day mortality, in-hospital mortality, microbiological clearance	8
Guanhao Zheng (29)	2022	China	82/82	CRKP	mROS	2019–2021	All site	Ceftazidime/Avibactam	PMB was administered at a dose of 1.25–1.5 mg/kg every 12 h; Ceftazidime/Avibactam 2.5 g was administered every 8 h	30-day mortality, microbiological clearance	8
Kang Chang (30)	2022	China	191/173	CRO	mROS	2018–2020	All site	Tigecycline	NA	28-day mortality, nephrotoxicity	7
Darowan S. Akajagbor (15)	2013	USA	67/106	MDR PA, AB	sROS	2008–2010	All site	Colistin	PMB was dosed at 15000–25000 units/kg/day over 24 h. Colistin was dosed at 5 mg/kg/day of ideal body weight (IBW) every 12 h	nephrotoxicity	9
Ritesh Aggarwal (16)	2018	India	51/61	KP, AB, *E. coli*	sPOS	2016–2017	All site	Colistin	Mean daily dose of PMB was 200 (IQR 180–240) mg; Mean daily dose of colistin was 233.3 (IQR 150–300) mg	nephrotoxicity	8
Michael J. Satlin (31)	2011	USA	25/62	CRKP	sROS	2005–2010	Urinary tract	Tigecycline and Aminoglycoside	Median daily doses of PMB was 2.25 (1.1–3.3) mg/kg of body weight/day	30-day mortality, Nephrotoxicity, microbiological clearance	8
Júlia Coelho França Quintanilha (32)	2019	Brazil	51/51	GNB	sROS	2010–2013	All site	Colistin	Average daily dose of PMB was1.5 ± 0.4*10^6^ IU; Average daily dose of Colistin was 3.3 ± 1.5*10^6^ IU	30-day mortality, Nephrotoxicity	9
Jiale Wang (33)	2023	China	52/28	CR-GNB	sROS	2021–2022	All site	Colistin	The duration for PMB was 9 (3–32) days, while for colistin was 11 (3–25) days	28-day mortality, Clinical success on day 7, microbiological clearance	9
Tiantian Tang (34)	2023	China	105/167	CRO	sROS	2020–2021	All site	Others	The maintenance dose of PMB was 40–100 mg every 12 h	30-day mortality, Nephrotoxicity	7
Shaohua Liu (35)	2023	China	121/83	CR-GNB	sROS	2021–2022	All site	Others	NA	28-day mortality, Nephrotoxicity	7
Jing Yang (36)	2022	China	393/352	CR-GNB	sROS	2018–2022	All site	Others	NA	30-day mortality, Nephrotoxicity	7
Junyan Qu (31)	2023	China	266/112	CR-GNB	sROS	2018–2022	All site	Ceftazidime/Avibactam	Dose of PMB was 50–75 mg, every 12 h; Dose of Ceftazidime/Avibactam was 2.5 g, every 8 h	In-hospital mortality, 28-day in-hospital mortality	8
Jionghe Wu (37)	2020	China	20/24	KP, AB, *E. coli*	sROS	2017–2019	Respiratory	Tigecycline	Daily dose of PMB was 0.75–1*10^6^ IU; Dose of tigecycline was 50 mg every 12 h	30-day mortality, microbiological clearance, the effective rate	9
Lei Zha (19)	2023	China	68/94	CRAB, CRKP	sROS	2019–2021	Respiratory	Tigecycline	Dose of PMB was 0.5–0.75*10^6^ IU, every 12 h	14-day mortality, microbiological clearance, Nephrotoxicity	7

sPOS, single-center prospective observational study; sROS, single-center retrospective observational study; mROS, multi-center retrospective observational study; mPOS, multi-center prospective observational study; NOS, Newcastle–Ottawa Scale. MU, million units; PMB, Polymyxin B; MIU, 1 million IU. Others, Other antibiotics (colistin; cefepime; imipenem; ciprofloxacin; meropenem; ceftazidime; aztreonam; piperacillin/tazobactam; tigecycline; cefoperazone/sulbactam; piperacillin/tazobactam; ceftazidime/Avibactam); AB, *Acinetobacter baumannii*; PA, *Pseudomonas aeruginosa*; KP, *Klebsiella pneumoniae*; *E. coli*, *Escherichia coli*; E. spp, Enterobacter spp; CRAB, Carbapenem-resistant *Acinetobacter baumannii*; CRPA, Carbapenem-resistant *Pseudomonas aeruginosa*; CRKP, Carbapenem-resistant *Klebsiella pneumoniae*; CRO, Carbapenem-resistant organism; CR-GNB, Carbapenem-resistant Gram-negative bacteria; GNB, Gram-negative bacteria; MDR, Multidrug resistant.

**Figure 2:**
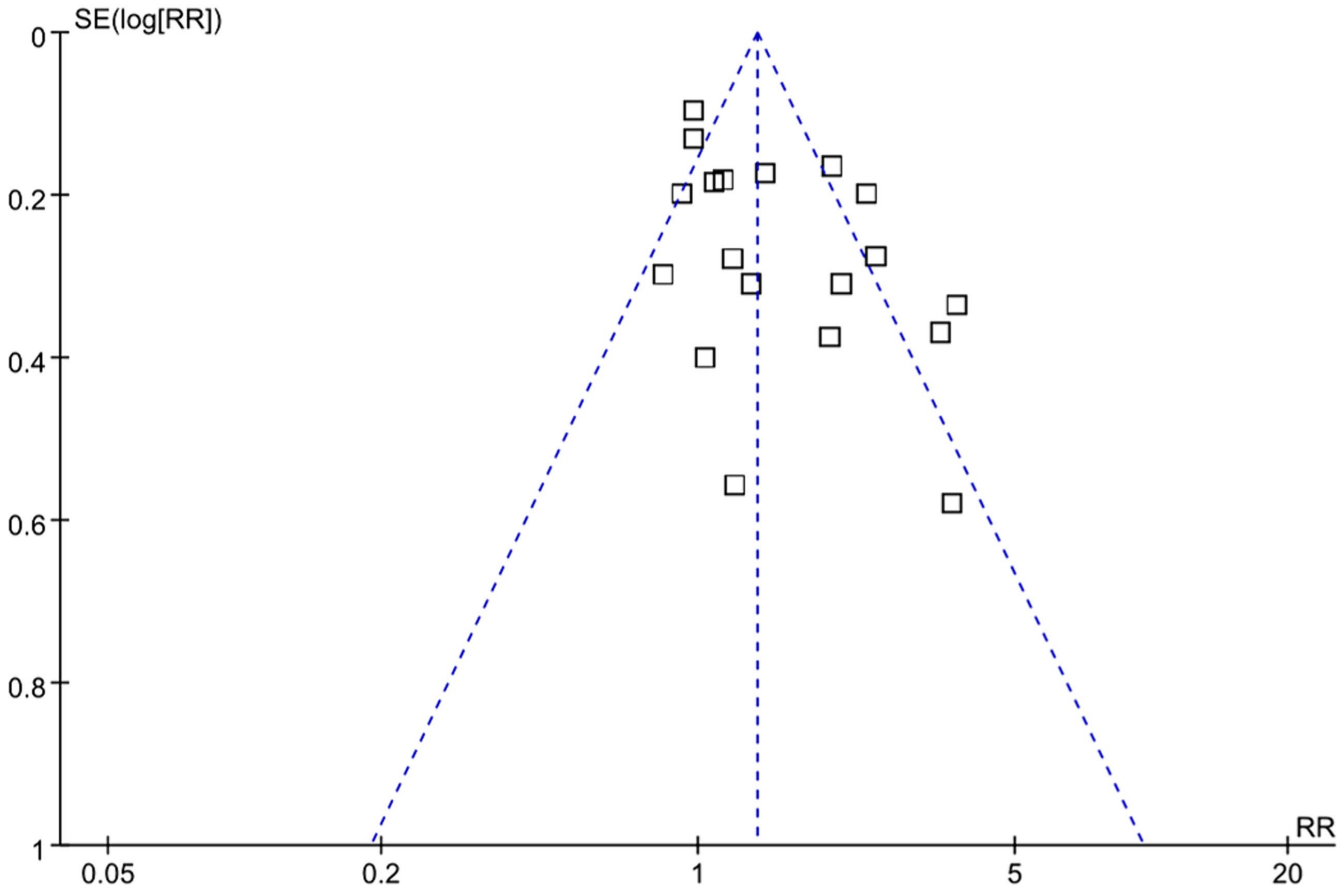
Funnel plot of mortality.

According to NOS analysis, quality scores ranged from 6 to 9, and 7 trials were rated as low risk (equal 9 score), 7 trials were rated as moderate risk (equal 8 score), and 8 trials were rated as high risk (equal 7 score or less than 7 score). The risk bias of included studies was shown in Table 1 and Supplementary Table S2. Visual analysis of the funnel plots suggested no publication bias (Figure 2).

### Mortality

**Figure 3:**
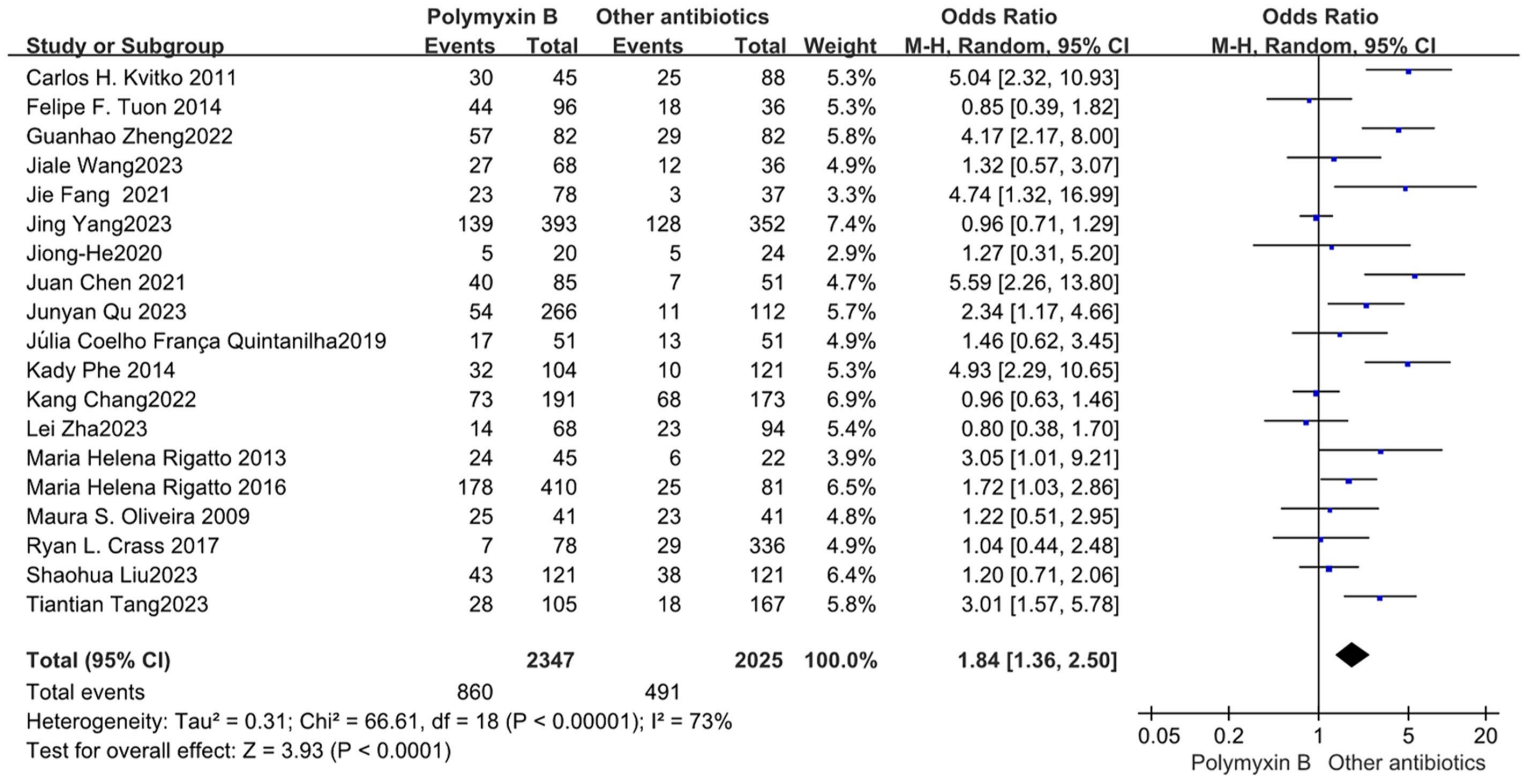
The mortality was compared between PMB and other antibiotics.

**Figure 4:**
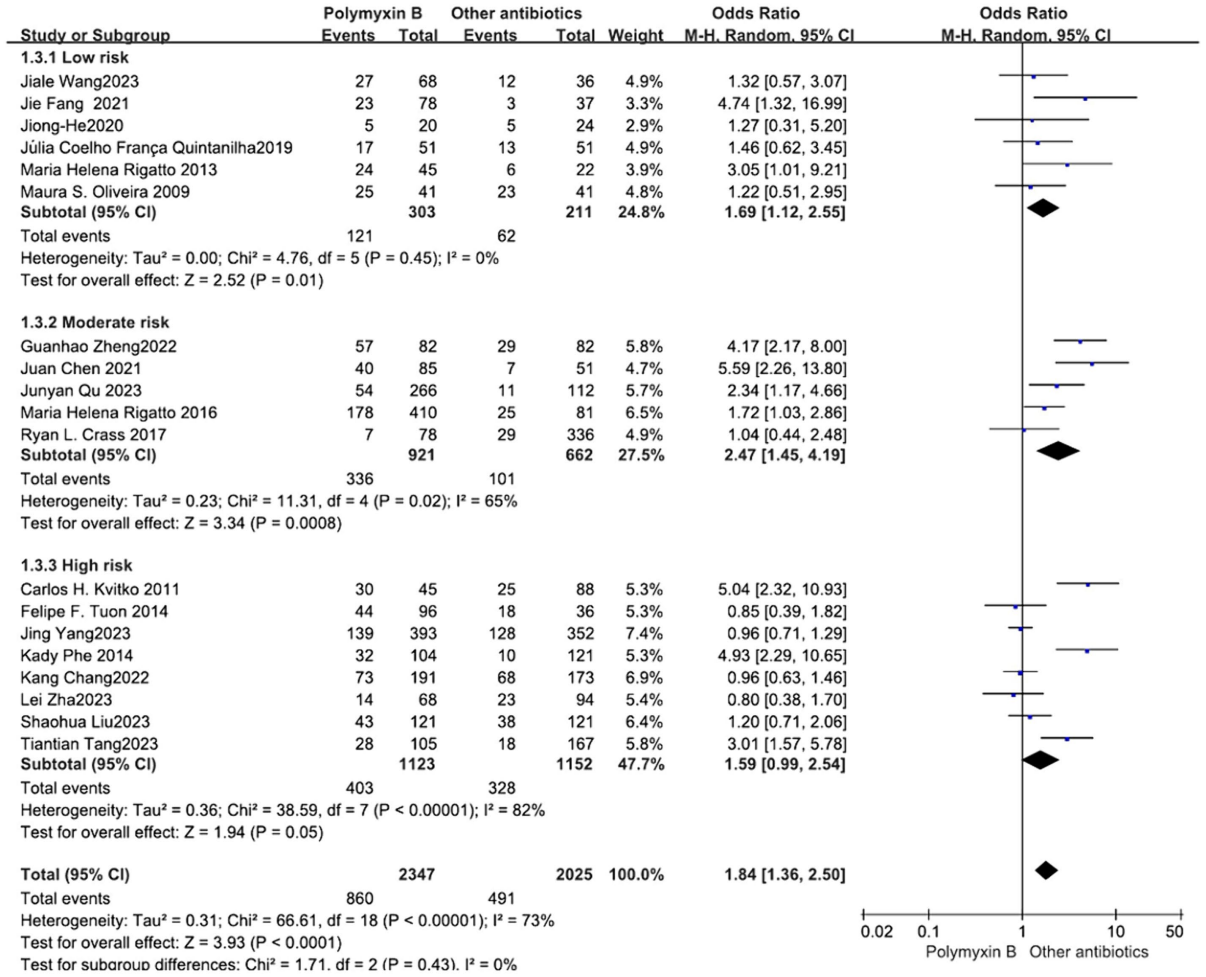
The mortality was compared between PMB and other antibiotics (subgroup analysis based on the risk of bias).

All included 19 trials (12–14, 19, 23–30, 32–38) with 4,372 patients totally mentioned the overall mortality, and our meta-analysis indicated that the mortality was higher in the PMB group than the control group (odds ratio 1.84, 95% confidence interval 1.36–2.50; *p*<0.00001; *I*^2^ = 73%, Figure 3). Subgroup analysis stratified by risk level indicates that the control group shows lower mortality rates in the low-risk level, along with reduced heterogeneity (odds ratio 1.69, 95% confidence interval 1.12–2.55; *p* = 0.01; *I*^2^ = 0%, Figure 4).

**Figure 5:**
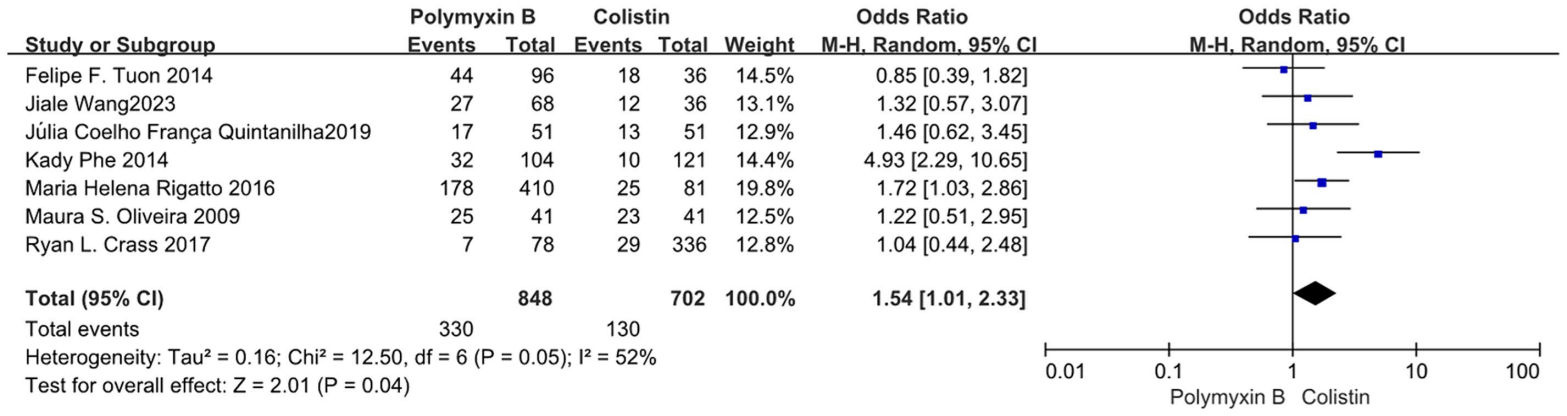
The mortality was compared between PMB and colistin.

**Figure 6:**
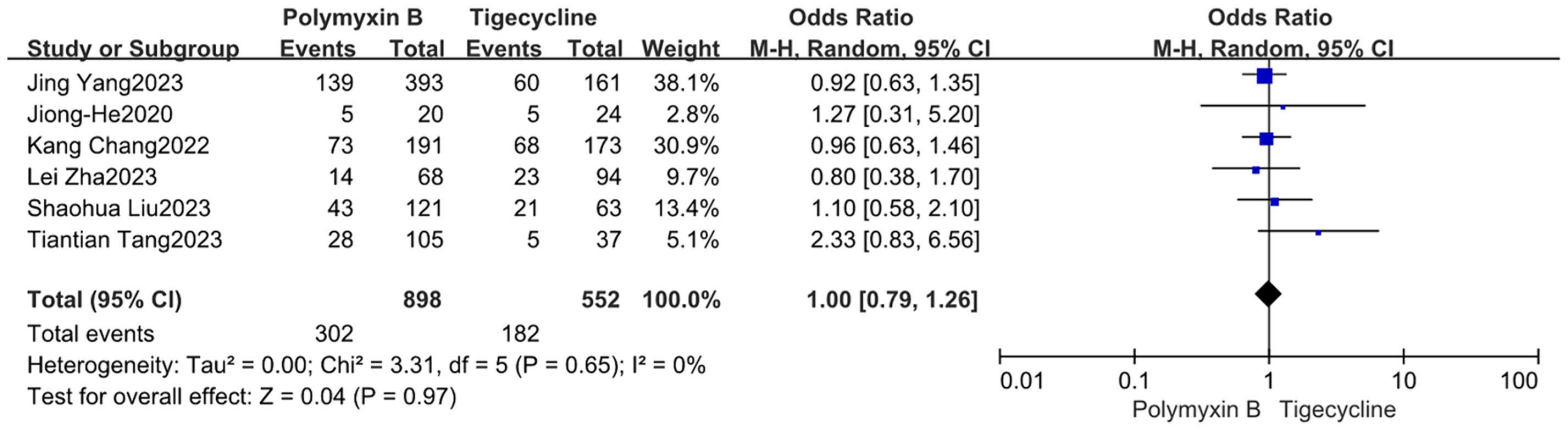
The mortality was compared between PMB and tigecycline.

**Figure 7:**
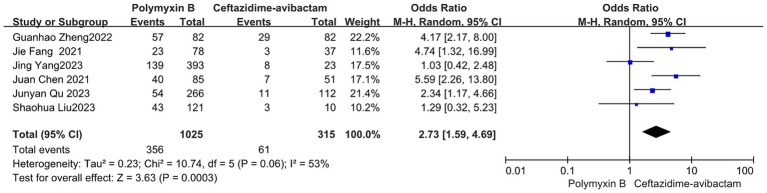
The mortality was compared between PMB and ceftazidime-avibactam.

**Figure 8:**
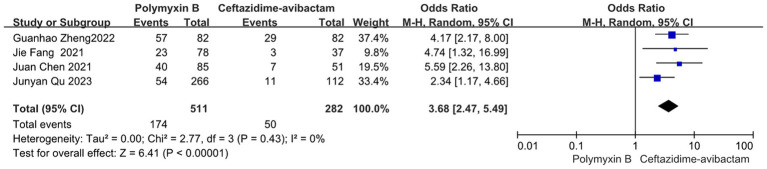
The mortality was compared between PMB and ceftazidime-avibactam (low and moderate risk of studies).

Our findings revealed that the colistin group exhibited a slightly lower mortality rate compared to the PMB group, but with high heterogeneity (odds ratio 1.54, 95% confidence interval 1.01–2.33; *p* = 0.04; I^2^ = 52%, Figure 5). Meanwhile, no significant difference in mortality was observed between the PMB group and the tigecycline group (odds ratio 1.00, 95% confidence interval 0.79–1.26; *p* = 0.65; *I*^2^ = 0%, Figure 6). However, the ceftazidime-avibactam group demonstrated a distinct advantage with lower mortality rates, despite still exhibiting high heterogeneity (odds ratio 2.73, 95% confidence interval 1.59–4.69; *p* = 0.0003; *I*^2^ = 53%, Figure 7). Notably, after excluding high-risk studies, we obtained consistent stronger results with improved heterogeneity (odds ratio 3.68, 95% confidence interval 2.47–5.49; *p*<0.00001; *I*^2^ = 0%, Figure 8).

### Nephrotoxicity

**Figure 9:**
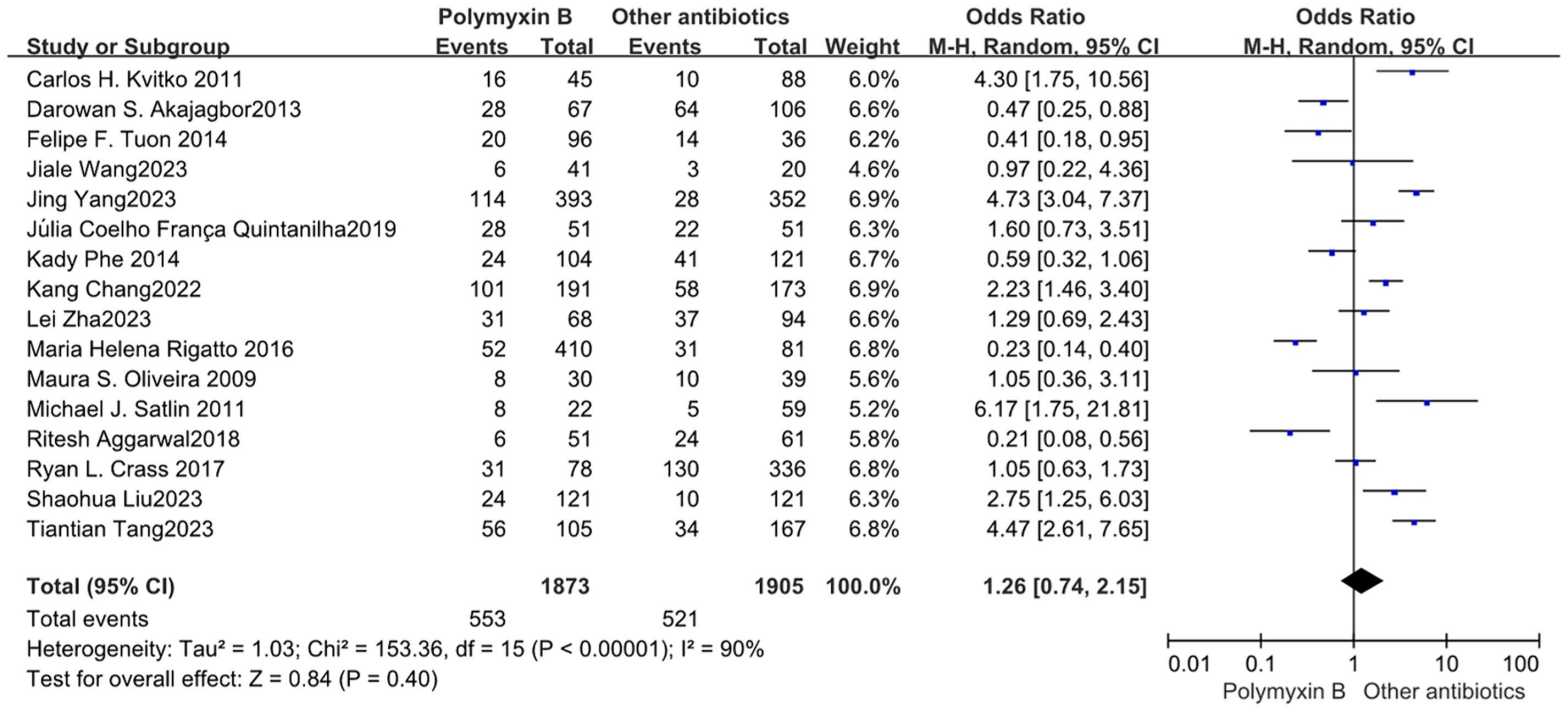
The nephrotoxicity was compared between PMB and other antibiotics.

**Figure 10:**
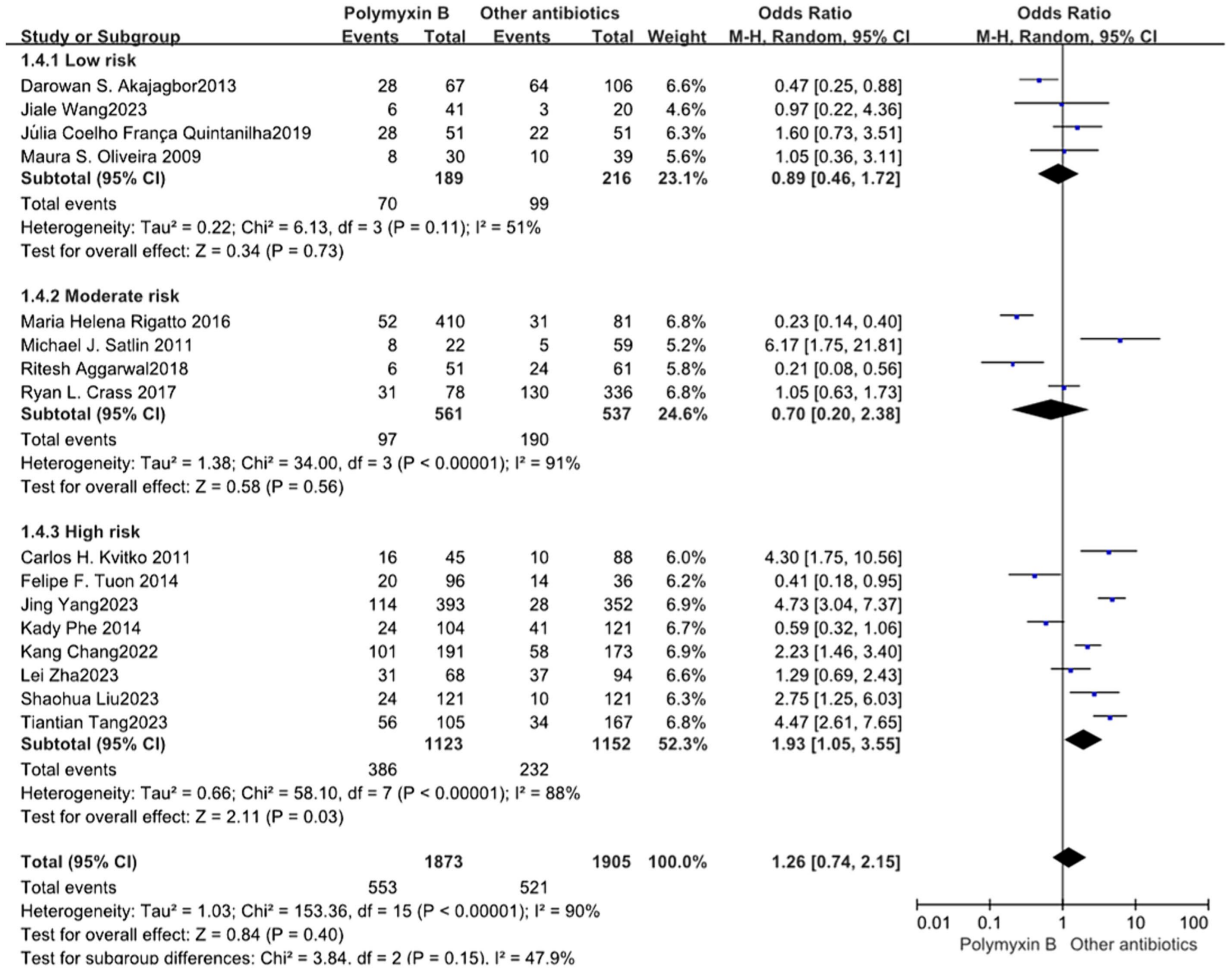
The nephrotoxicity was compared between PMB and other antibiotics (subgroup analysis based on the risk of bias).

Our meta-analysis included a total of 16 trials (12–16, 19, 23, 24, 26, 30–36) involving 3,778 patients, all of which reported nephrotoxicity. The results revealed no significant difference in nephrotoxicity between the PMB group and the control group with high heterogeneity (odds ratio: 1.26, 95% confidence interval: 0.74–2.15; *p*<0.40; *I*^2^ = 90%, Figure 9). Stratifying the subgroup analysis by risk level yielded similar results but did not alleviate heterogeneity (Figure 10).

**Figure 11:**
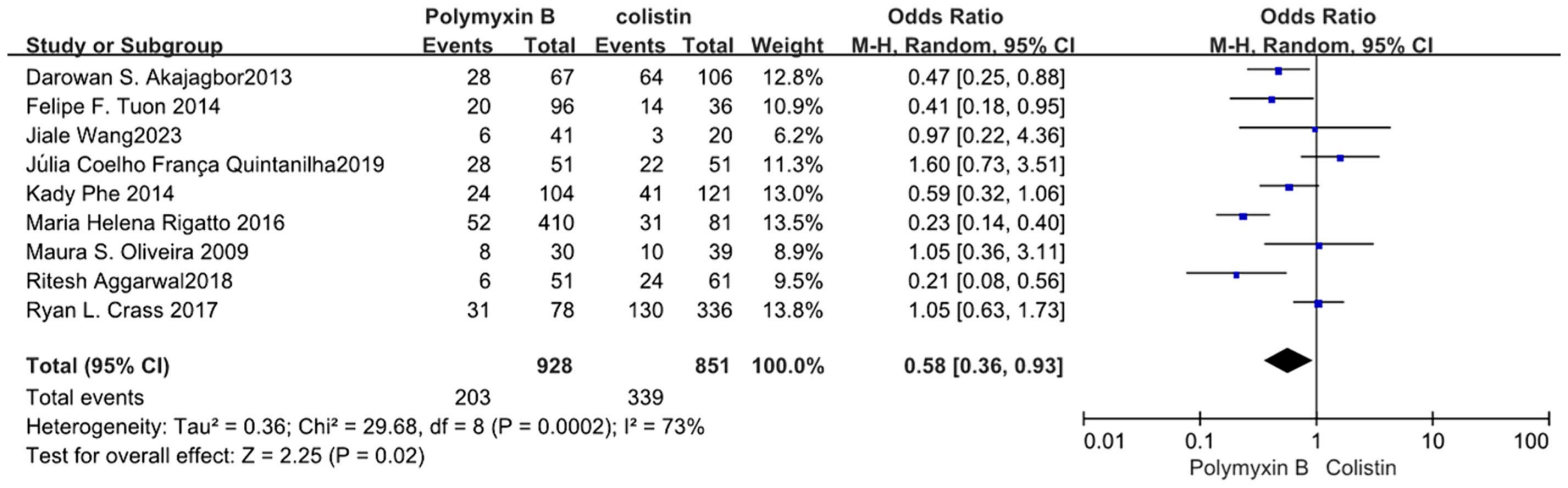
The nephrotoxicity was compared between PMB and colistin.

**Figure 12:**
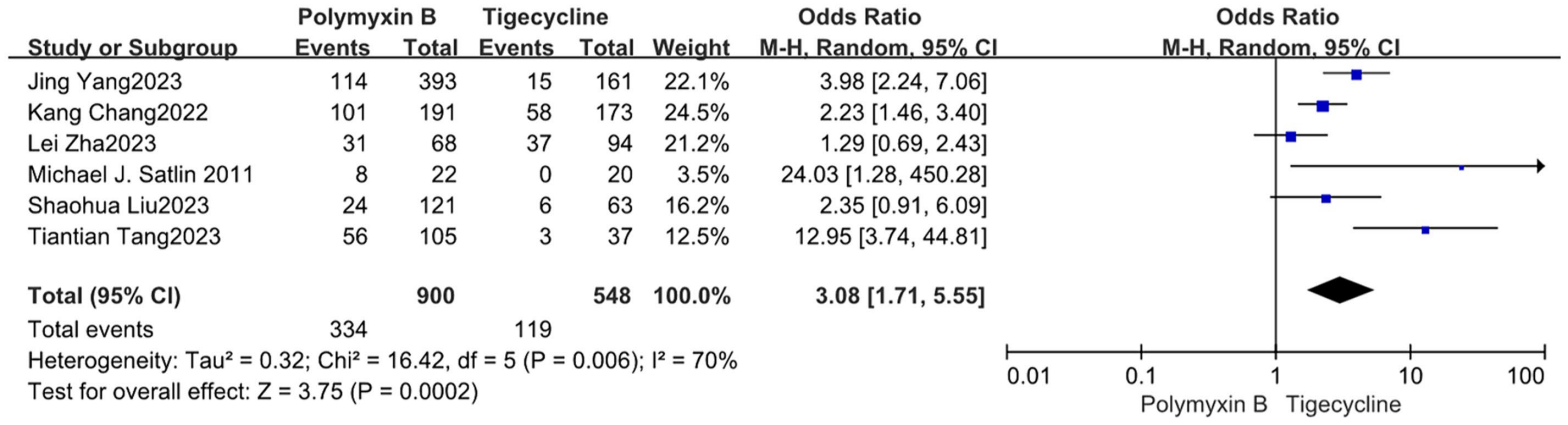
The nephrotoxicity was compared between PMB and tigecycline.

We found that the PMB group had a lower nephrotoxicity rate compared to the colistin group but exhibited high heterogeneity in the results (odds ratio 0.58, 95% CI 0.36–0.93; *p* = 0.02; *I*^2^ = 73%, Figure 11). Conversely, the tigecycline group had a lower nephrotoxicity rate, despite similarly displaying high heterogeneity (odds ratio 3.08, 95% CI 1.71–5.55; *p* = 0.0002; *I*^2^ = 70%, Figure 12).

### Bacterial clearance

**Figure 13:**
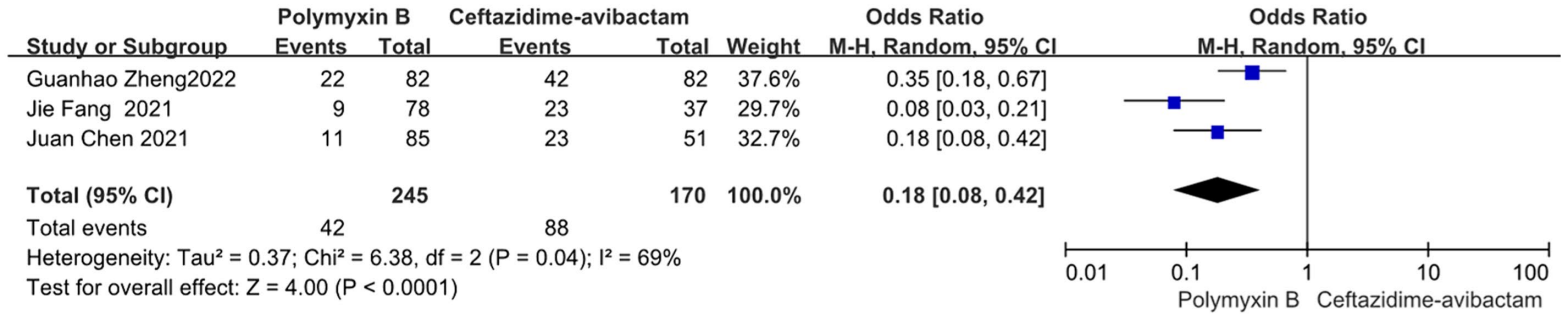
The bacterial clearance rate was compared between PMB and ceftazidime-avibactam.

Compared to the PMB group, the ceftazidime-avibactam group demonstrated better bacterial clearance (odds ratio 0.18, 95% CI 0.08–0.42; *p*<0.0001; *I*^2^ = 69%, Figure 13).

## Discussion

This is a systematic review and meta-analysis of 22 clinical trials to evaluate the effectiveness and safety of PMB in patients with nosocomial infections. Our meta-analysis revealed that PMB is not superior to other antibiotics in terms of mortality, specifically when compared to ceftazidime-avibactam. However, PMB demonstrated an advantage in terms of nephrotoxicity compared to colistin.

Due to the rise of pathogen resistance, there is a need for the development of novel antibiotics and strategies. Despite the availability of new antimicrobial agents, the issue of their high cost persists. It is of paramount importance to reevaluate old antibiotics and investigate their clinical efficacy. Our meta-analysis revealed that PMB is not superior to other antibiotics in terms of mortality, particularly in comparison to ceftazidime-avibactam. Therefore, ceftazidime-avibactam could be a favorable option for nosocomial infections. However, the cost-effectiveness of the comparing antibiotics is not addressed in the present study. Moreover, PMB serves as an invaluable salvage therapy and should be contemplated for combination therapy. Naturally, additional evidence and further qualified studies are necessary to ascertain the cost-efficacy of PMB usage.

Both PMB and colistin are commonly administered in combination with other antibiotics to enhance effectiveness and reduce resistance development. In Maura S. Oliveira’s study, both the PMB and colistin groups received combined antibiotics, including amphotericin, ampicillin, carbapenem, cephalosporin, ciprofloxacin, metronidazole, piperacillin-tazobactam, trimethoprim-sulfamethoxazole, and vancomycin, with no statistically significant differences observed (23). Similar results were also seen in Ryan L. Crass’s study (26). All the studies included were retrospective. The extensive range of antibiotics used in both groups limited further statistical analysis. The variety of antibiotics used in combination may potentially impact outcomes, underscoring the need for further research to investigate the efficacy of antibiotic combination therapy.

Our meta-analysis indicated that PMB demonstrated an advantage in terms of nephrotoxicity compared to colistin. Therefore, PMB may be a more favorable choice than colistin for treating nosocomial infections in patients with renal insufficiency. Additionally, the nebulized administration of both PMB and colistin has been investigated (11, 39–44). While we acknowledge the association between drug dosage and nephrotoxicity, the PMB dosages in different studies vary in presentation. Some articles specify dosages based on unit weight, while others mention total daily dosages. This inconsistency in reporting methods hinders the feasibility of performing regression analysis to explore the relationship between PMB dosage and nephrotoxicity. To explore new potential applications for old antibiotics, such as PMB and colistin, further research is warranted to investigate appropriate selection criteria, administration methods, and dosage recommendations.

The majority of the included articles are from Brazil and China. Subgroup analysis was also performed (Supplementary Figures S1, S2), revealing that there was no significant difference in nephrotoxicity between the PMB group and the control group in Brazil and the USA. However, the PMB group exhibited a higher risk of developing nephrotoxicity when compared to the control group. The possible reason for this observation could be that many studies included from China in recent years predominantly used tigecycline and tigecycline as comparator groups. PMB shows stronger nephrotoxicity compared to these two drugs. In earlier studies, colistin was the primary comparator, and there was minimal difference in nephrotoxicity.

A few limitations were still present in our study. First, all the studies included in this analysis were cohort studies, and the level of evidence was very low. Second, there is variability in the timing of mortality among different studies. Most studies assessed 30-day mortality, while others focused on in-hospital mortality, 28-day mortality, and 14-day mortality. Such heterogeneity may potentially impair the quality of the synthesized evidence. Third, nosocomial infections often require the combined application of antibiotics. However, our study did not account for the concurrent use of medications in each group. Finally, there was significant heterogeneity in the dosage and duration of antibiotic between studies for many of our outcomes. Hence, our current findings were weakened, and our study was downgraded one level.

## Conclusion

Our meta-analysis showed that PMB is not superior to other antibiotics in terms of mortality, specifically when compared to ceftazidime-avibactam. However, PMB demonstrated an advantage in terms of nephrotoxicity compared to colistin. Additionally, there is a need for further high-quality studies to elucidate the appropriate antibiotic selection for nosocomial infections.

## Data availability statement

The raw data supporting the conclusions of this article will be made available by the authors, without undue reservation.

## Ethics statement

Ethical approval was not required for the study involving humans in accordance with the local legislation and institutional requirements. Written informed consent to participate in this study was not required from the participants or the participants’ legal guardians/next of kin in accordance with the national legislation and the institutional requirements.

## Author contributions

LP: Writing – original draft, Software, Methodology, Formal analysis, Data curation. ZZ: Writing – original draft, Funding acquisition. XQ: Writing – original draft, Data curation. YZ: Writing – original draft, Project administration, Data curation. TS: Writing – review & editing, Resources, Investigation. LC: Writing – original draft, Validation, Investigation. JZ: Writing – original draft, Resources, Methodology, Conceptualization. XL: Writing – original draft, Validation, Methodology, Data curation. PM: Writing – review & editing, Visualization, Supervision, Resources, Conceptualization.

## Supplementary material

Supplementary FIGURE S1 The mortality was compared between PMB and other antibiotics (subgroup analysis based on different countries).

Supplementary FIGURE S2 The nephrotoxicity was compared between PMB and other antibiotics (subgroup analysis based on different countries).

Supplementary Table S1 Search strategy.

Supplementary Table S2 Quality evaluation of the eligible studies with Newcastle-Ottawa scale.
