# Supplementary material for: Efficiency of polymyxin B treatment against nosocomial infection: a systematic review and meta-analysis
Source: Front Med (Lausanne). 2024 May 28;11:1400757. doi: 10.3389/fmed.2024.1400757 (PMC11165566; doi:10.3389/fmed.2024.1400757)
Supplement: Supplementary FIGURE S1 — The mortality was compared between PMB and other antibiotics (subgroup analysis based on different countries). [file Data_Sheet_1.DOCX]

**Table S1. Search strategy**

|  | **PUBMED** |  |
| --- | --- | --- |
| #1 | Polymyxin B[Mesh] | 3,659 |
| #2 | (Polymyxin B[Text Word]) OR (Polymyxin-B[Text Word]) OR (Polymyxin B Sulfate[Text Word]) OR (Aerosporin[Text Word]) | 7,056 |
| #3 | #1 OR #2 | 7,056 |
| #4 | "Drug Resistance, Multiple, Bacterial"[Mesh] | 25,564 |
| #5 | (multi-resistance bacterial infection[Text Word]) OR (Drug Resistance, Multiple, Bacterial[Text Word]) OR (Hospital Infection[Text Word]) OR (Healthcare Associated Infections[Text Word]) OR (Healthcare Associated Infection[Text Word]) OR (Cross Infections[Text Word]) OR (Nosocomial Infection[Text Word]) OR (Nosocomial Infections[Text Word]) OR (Hospital Infections[Text Word]) OR (Hospital-Acquired Pneumonia[Text Word]) OR ( Pseudomonas aeruginosa Infection[Text Word]) OR (Carbapenem-Resistant Klebsiella Infections[Text Word]) OR (Acinetobacter baumannii infection[Text Word]) OR ( carbapenem-resistant gram-negative bacteria[Text Word]) OR (Carbapenem resistant Enterobacteriaceae bacteria[Text Word]) OR (vancomycin-resistant enterococci[Text Word]) OR (Multiple Antibacterial Drug Resistance[Text Word]) | 57,884 |
| #6 | #4 OR #5 | 57,884 |
| #7 | Mortality[Mesh] | 423,895 |
| #8 | (Mortalities[Text Word]) OR (Case Fatality Rate[Text Word]) OR (Case Fatality Rates[Text Word]) OR (Rate, Case Fatality[Text Word]) OR (Rates, Case Fatality[Text Word]) OR (CFR Case Fatality Rate[Text Word]) OR (Crude Death Rate[Text Word]) OR (Crude Death Rates[Text Word]) OR (Death Rate, Crude[Text Word]) OR (Rate, Crude Death[Text Word]) OR (Crude Mortality Rate[Text Word]) OR (Crude Mortality Rates[Text Word]) OR (Mortality Rate, Crude[Text Word]) OR (Rate, Crude Mortality[Text Word]) OR (Death Rate[Text Word]) OR (Death Rates[Text Word]) OR (Rate, Death[Text Word]) OR (Mortality Rate[Text Word]) OR (Mortality Rates[Text Word]) OR (Rate, Mortality[Text Word]) OR (Mortality, Excess[Text Word]) OR (Excess Mortality[Text Word]) OR (Excess Mortalities[Text Word]) OR (Decline, Mortality[Text Word]) OR (Mortality Declines[Text Word]) OR (Mortality Decline[Text Word]) OR (Mortality Determinants[Text Word]) OR (Determinants, Mortality[Text Word]) OR (Determinant, Mortality[Text Word]) OR (Mortality Determinant[Text Word]) OR (Mortality, Differential[Text Word]) OR (Differential Mortality[Text Word]) OR (Differential Mortalities[Text Word]) OR (Age-Specific Death Rate[Text Word]) OR (Death Rate, Age-Specific[Text Word]) OR (Age-Specific Death Rates[Text Word]) OR (Rate, Age-Specific Death[Text Word]) OR (Age Specific Death Rate[Text Word]) OR (Nephrotoxicity [Text Word]) OR (renal injury[Text Word]) OR (Renal trauma[Text Word]) OR (Acute Kidney Injury[Text Word]) OR (Renal Failure[Text Word]) OR (Acute Renal Failure[Text Word]) OR (Acute Kidney Insufficiencies[Text Word]) OR (renal toxicity[Text Word]) OR ( Kidney Injuries[Text Word]) OR (Renal Injury[Text Word]) OR (Kidney Injury[Text Word]) OR ( Renal Injury[Text Word]) OR ( Renal Injuries[Text Word]) OR (Renal Insufficiency[Text Word]) OR ( Renal Insufficiencies[Text Word]) OR (Renal Insufficiencies[Text Word]) OR ( Renal Insufficiency[Text Word]) OR (Kidney Insufficiency[Text Word]) OR ( Kidney Insufficiencies[Text Word]) OR (Kidney Insufficiencies[Text Word]) OR (Kidney Failure[Text Word]) OR (Kidney Failures[Text Word]) OR (Renal Failure[Text Word]) OR (Renal Failures[Text Word]) | 530,546 |
| #9 | #7 OR #8 | 889,551 |
| #10 | #3 AND #6 AND #9 | 79 |

|  | **Embase** |  |
| --- | --- | --- |
| #1 | 'polymyxin B'/exp | 13110 |
| #2 | 'Polymyxin B':ab,ti or 'Polymyxin-B':ab,ti or 'Polymyxin B Sulfate':ab,ti or 'Aerosporin':ab,ti | 7127 |
| #3 | #1 OR #2 | 15183 |
| #4 | 'multidrug resistance'/exp | 55846 |
| #5 | 'multi-resistance bacterial infection':ab,ti OR 'drug resistance, multiple, bacterial':ab,ti OR 'hospital infection':ab,ti OR 'healthcare associated infections':ab,ti OR 'healthcare associated infection':ab,ti OR 'cross infections':ab,ti OR 'nosocomial infection':ab,ti OR 'nosocomial infections':ab,ti OR 'hospital infections':ab,ti OR 'hospital-acquired pneumonia':ab,ti OR 'pseudomonas aeruginosa infection':ab,ti OR 'carbapenem-resistant klebsiella infections':ab,ti OR 'acinetobacter baumannii infection':ab,ti OR 'carbapenem-resistant gram-negative bacteria':ab,ti OR 'carbapenem resistant enterobacteriaceae bacteria':ab,ti OR 'vancomycin-resistant enterococci':ab,ti OR 'multiple antibacterial drug resistance':ab,ti | 44654 |
| #6 | #4 OR #5 | 98144 |
| #7 | 'mortality'/exp | 1,397,330 |
| #8 | 'mortalities':ab,ti OR 'case fatality rate':ab,ti OR 'case fatality rates':ab,ti OR 'rate, case fatality':ab,ti OR 'rates, case fatality':ab,ti OR 'cfr case fatality rate':ab,ti OR 'crude death rate':ab,ti OR 'crude death rates':ab,ti OR 'death rate, crude':ab,ti OR 'rate, crude death':ab,ti OR 'crude mortality rate':ab,ti OR 'crude mortality rates':ab,ti OR 'mortality rate, crude':ab,ti OR 'rate, crude mortality':ab,ti OR 'death rate':ab,ti OR 'rate, death':ab,ti OR 'death rates':ab,ti OR 'mortality rate':ab,ti OR 'mortality rates':ab,ti OR 'rate, mortality':ab,ti OR 'mortality, excess':ab,ti OR 'excess mortality':ab,ti OR 'excess mortalities':ab,ti OR 'decline, mortality':ab,ti OR 'mortality declines':ab,ti OR 'mortality decline':ab,ti OR 'mortality determinants':ab,ti OR 'determinants, mortality':ab,ti OR 'determinant, mortality':ab,ti OR 'mortality determinant':ab,ti OR 'mortality, differential':ab,ti OR 'differential mortality':ab,ti OR 'differential mortalities':ab,ti OR 'age-specific death rates':ab,ti OR 'age-specific death rate':ab,ti OR 'death rate, age-specific':ab,ti OR 'rate, age-specific death':ab,ti OR 'age specific death rate':ab,ti OR 'nephrotoxicity':ab,ti OR 'renal injury':ab,ti OR 'renal trauma':ab,ti OR 'acute kidney injury':ab,ti OR 'acute renal failure':ab,ti OR 'acute kidney insufficiencies':ab,ti OR 'renal toxicity':ab,ti OR 'kidney injuries':ab,ti OR 'kidney injury':ab,ti OR 'renal injury':ab,ti OR ' renal injury':ab,ti OR ' renal injuries':ab,ti OR 'renal insufficiency':ab,ti OR ' renal insufficiencies':ab,ti OR 'renal insufficiencies':ab,ti OR ' renal insufficiency':ab,ti OR 'kidney insufficiency':ab,ti OR 'kidney insufficiencies':ab,ti OR 'kidney failure':ab,ti OR 'kidney failures':ab,ti OR 'renal failure':ab,ti OR 'renal failures':ab,ti | 1006858 |
| #9 | #6 OR #7 | 1922178 |
| #10 | #3 AND #6 AND #9 | 258 |

|  | **Cochranelibrary** |  |
| --- | --- | --- |
| #1 | (Polymyxin B):ti,ab,kw OR (Polymyxin-B):ti,ab,kw OR (Polymyxin B Sulfate):ti,ab,kw OR (Aerosporin):ti,ab,kw | 407 |
| #2 | (multi-resistance bacterial infection):ti,ab,kw OR (Drug Resistance, Multiple, Bacterial):ti,ab,kw OR (Hospital Infection):ti,ab,kw OR (Healthcare Associated Infections):ti,ab,kw OR (Healthcare Associated Infection):ti,ab,kw OR (Cross Infections):ti,ab,kw OR (Nosocomial Infection):ti,ab,kw OR (Nosocomial Infections):ti,ab,kw OR (Hospital Infections):ti,ab,kw OR (Hospital-Acquired Pneumonia):ti,ab,kw OR ( Pseudomonas aeruginosa Infection):ti,ab,kw OR (Carbapenem-Resistant Klebsiella Infections):ti,ab,kw OR (Acinetobacter baumannii infection):ti,ab,kw OR ( carbapenem-resistant gram-negative bacteria):ti,ab,kw OR (Carbapenem resistant Enterobacteriaceae bacteria):ti,ab,kw OR (vancomycin-resistant enterococci):ti,ab,kw OR (Multiple Antibacterial Drug Resistance):ti,ab,kw OR | 27844 |
| #3 | (Mortalities):ti,ab,kw OR (Case Fatality Rate):ti,ab,kw OR (Case Fatality Rates):ti,ab,kw OR (Rate, Case Fatality):ti,ab,kw OR (Rates, Case Fatality):ti,ab,kw OR (CFR Case Fatality Rate):ti,ab,kw OR (Crude Death Rate):ti,ab,kw OR (Crude Death Rates):ti,ab,kw OR (Death Rate, Crude):ti,ab,kw OR (Rate, Crude Death):ti,ab,kw OR (Crude Mortality Rate):ti,ab,kw OR (Crude Mortality Rates):ti,ab,kw OR (Mortality Rate, Crude):ti,ab,kw OR (Rate, Crude Mortality):ti,ab,kw OR (Death Rate):ti,ab,kw OR (Death Rates):ti,ab,kw OR (Rate, Death):ti,ab,kw OR (Mortality Rate):ti,ab,kw OR (Mortality Rates):ti,ab,kw OR (Rate, Mortality):ti,ab,kw OR (Mortality, Excess):ti,ab,kw OR (Excess Mortality):ti,ab,kw OR (Excess Mortalities):ti,ab,kw OR (Decline, Mortality):ti,ab,kw OR (Mortality Declines):ti,ab,kw OR (Mortality Decline):ti,ab,kw OR (Mortality Determinants):ti,ab,kw OR (Determinants, Mortality):ti,ab,kw OR (Determinant, Mortality):ti,ab,kw OR (Mortality Determinant):ti,ab,kw OR (Mortality, Differential):ti,ab,kw OR (Differential Mortality):ti,ab,kw OR (Differential Mortalities):ti,ab,kw OR (Age-Specific Death Rate):ti,ab,kw OR (Age-Specific Death Rates):ti,ab,kw OR (Rate, Age-Specific Death):ti,ab,kw OR (Death Rate, Age-Specific):ti,ab,kw OR (Age Specific Death Rate):ti,ab,kw OR (Nephrotoxicity ):ti,ab,kw OR (renal injury):ti,ab,kw OR (Renal trauma):ti,ab,kw OR (Acute Kidney Injury):ti,ab,kw OR (Renal Failure):ti,ab,kw OR (Acute Renal Failure):ti,ab,kw OR (Acute Kidney Insufficiencies):ti,ab,kw OR (renal toxicity):ti,ab,kw OR ( Kidney Injuries):ti,ab,kw OR (Kidney Injury):ti,ab,kw OR (Renal Injury):ti,ab,kw OR ( Renal Injury):ti,ab,kw OR (Renal Insufficiency):ti,ab,kw OR ( Renal Injuries):ti,ab,kw OR ( Renal Insufficiencies):ti,ab,kw OR (Renal Insufficiencies):ti,ab,kw OR ( Renal Insufficiency):ti,ab,kw OR (Kidney Insufficiency):ti,ab,kw OR ( Kidney Insufficiencies):ti,ab,kw OR (Kidney Insufficiencies):ti,ab,kw OR (Kidney Failure):ti,ab,kw OR (Kidney Failures):ti,ab,kw OR (Renal Failure):ti,ab,kw OR (Renal Failures):ti,ab,kw | 112882 |
| #4 | #1 AND #2 AND #3 | 30 |

|  | **Web of science** |  |
| --- | --- | --- |
| #1 | Polymyxin B or Polymyxin-B or Polymyxin B Sulfate or Aerosporin （topic） | 7072 |
| #2 | \| multi-resistance bacterial infection or Drug Resistance, Multiple, Bacterial or Hospital Infection or Healthcare Associated Infections or Healthcare Associated Infection or Cross Infections or Nosocomial Infections or Nosocomial Infection or Hospital Infections or Hospital-Acquired Pneumonia or Pseudomonas aeruginosa Infection or Carbapenem-Resistant Klebsiella Infections or Acinetobacter baumannii infection or carbapenem-resistant gram-negative bacteria or Carbapenem resistant Enterobacteriaceae bacteria or vancomycin-resistant enterococci or Multiple Antibacterial Drug Resistance （topic） \| \| --- \| | 325763 |
| #3 | Mortalities or Case Fatality Rate or Case Fatality Rates or Rate, Case Fatality or Rates, Case Fatality or CFR Case Fatality Rate or Crude Death Rate or Crude Death Rates or Death Rate, Crude or Rate, Crude Death or Crude Mortality Rate or Crude Mortality Rates or Mortality Rate, Crude or Rate, Crude Mortality or Death Rate or Death Rates or Rate, Death or Mortality Rate or Mortality Rates or Rate, Mortality or Mortality, Excess or Excess Mortality or Excess Mortalities or Decline, Mortality or Mortality Declines or Mortality Decline or Mortality Determinants or Determinants, Mortality or Determinant, Mortality or Mortality Determinant or Mortality, Differential or Differential Mortality or Differential Mortalities or Age-Specific Death Rate or Age-Specific Death Rates or Death Rate, Age-Specific or Rate, Age-Specific Death or Age Specific Death Rate or Nephrotoxicity or renal injury or Renal trauma or Acute Kidney Injury or Renal Failure or Acute Renal Failure or renal toxicity or Acute Kidney Insufficiencies or Kidney Injury or Kidney Injuries or Renal Injury or Renal Injury or Renal Injuries or Renal Insufficiency or Renal Insufficiencies or Renal Insufficiencies or Renal Insufficiency or Kidney Insufficiency or Kidney Insufficiencies or Kidney Insufficiencies or Kidney Failure or Kidney Failures or Renal Failure or Renal Failures （topic） | 1650583 |
| #4 | #1 AND #2 AND #3 | 465 |

**Table S2. Quality evaluation of the eligible studies with Newcastle-Ottawa scale.**

|  | Selection | | | | Comparability | outcome | | | score |
| --- | --- | --- | --- | --- | --- | --- | --- | --- | --- |
| Study | Representativeness | Selection of non-exposed | Ascertainment of exposure | Outcome not present at start | Comparability on most important factors or other risk factor | Assessment of outcome | Long enough follow-up | Adequacy（completeness）of follow-up |  |
| Maura S. Oliveira1 | 1 | 1 | 1 | 1 | 2 | 1 | 1 | 1 | 9 |
| Carlos H. Kvitko2 | 1 | 1 | 1 | 1 | 1 | 1 | 1 | 0 | 7 |
| Maria Helena Rigatto3 | 1 | 1 | 1 | 1 | 2 | 1 | 1 | 1 | 9 |
| Kady Phe4 | 1 | 1 | 1 | 1 | 1 | 1 | 1 | 0 | 7 |
| Felipe F. Tuon5 | 1 | 1 | 1 | 1 | 0 | 1 | 1 | 0 | 6 |
| Maria Helena Rigatto6 | 1 | 1 | 1 | 1 | 1 | 1 | 1 | 1 | 8 |
| Ryan L. Crass7 | 1 | 1 | 1 | 1 | 1 | 1 | 1 | 1 | 8 |
| Jie Fang 8 | 1 | 1 | 1 | 1 | 2 | 1 | 1 | 1 | 9 |
| Juan Chen9 | 1 | 1 | 1 | 1 | 1 | 1 | 1 | 1 | 8 |
| Guanhao Zheng10 | 1 | 1 | 1 | 1 | 1 | 1 | 1 | 1 | 8 |
| Kang Chang11 | 1 | 1 | 1 | 1 | 0 | 1 | 1 | 1 | 7 |
| Darowan S. Akajagbor12 | 1 | 1 | 1 | 1 | 2 | 1 | 1 | 1 | 9 |
| Ritesh Aggarwal13 | 1 | 1 | 1 | 1 | 1 | 1 | 1 | 1 | 8 |
| Michael J. Satlin14 | 1 | 1 | 1 | 1 | 1 | 1 | 1 | 1 | 8 |
| Júlia Coelho França Quintanilha 15 | 1 | 1 | 1 | 1 | 2 | 1 | 1 | 1 | 9 |
| Jiale Wang16 | 1 | 1 | 1 | 1 | 2 | 1 | 1 | 1 | 9 |
| Tiantian Tang17 | 1 | 1 | 1 | 1 | 0 | 1 | 1 | 1 | 7 |
| Shaohua Liu18 | 1 | 1 | 1 | 1 | 0 | 1 | 1 | 1 | 7 |
| Jing Yang19 | 1 | 1 | 1 | 1 | 0 | 1 | 1 | 1 | 7 |
| [Junyan Qu](http://pubmed.01.bt8.net/?term=Qu+J&cauthor_id=37247645)^20^ | 1 | 1 | 1 | 1 | 1 | 1 | 1 | 1 | 8 |
| Jiong-He^21^ | 1 | 1 | 1 | 1 | 2 | 1 | 1 | 1 | 9 |
| Lei Zha^22^ | 1 | 1 | 1 | 1 | 1 | 1 | 0 | 1 | 7 |

1. Oliveira MS, Prado GV, Costa SF, et al. Polymyxin B and colistimethate are comparable as to efficacy and renal toxicity. Diagn Microbiol Infect Dis. 2009;65(4):431-434.

2. Kvitko CH, Rigatto MH, Moro AL, et al. Polymyxin B versus other antimicrobials for the treatment of pseudomonas aeruginosa bacteraemia. J Antimicrob Chemother. 2011;66(1):175-179.

3. Rigatto MH, Ribeiro VB, Konzen D, et al. Comparison of polymyxin B with other antimicrobials in the treatment of ventilator-associated pneumonia and tracheobronchitis caused by Pseudomonas aeruginosa or Acinetobacter baumannii. Infection. 2013;41(2):321-328.

4. Phe K, Lee Y, McDaneld PM, et al. In vitro assessment and multicenter cohort study of comparative nephrotoxicity rates associated with colistimethate versus polymyxin B therapy. Antimicrob Agents Chemother. 2014;58(5):2740-2746.

5. Tuon FF, Rigatto MH, Lopes CK, et al. Risk factors for acute kidney injury in patients treated with polymyxin B or colistin methanesulfonate sodium. Int J Antimicrob Agents. 2014;43(4):349-352.

6. Rigatto MH, Oliveira MS, Perdigao-Neto LV, et al. Multicenter Prospective Cohort Study of Renal Failure in Patients Treated with Colistin versus Polymyxin B. Antimicrob Agents Chemother. 2016;60(4):2443-2449.

7. Crass RL, Rutter WC, Burgess DR, et al. Nephrotoxicity in Patients with or without Cystic Fibrosis Treated with Polymyxin B Compared to Colistin. Antimicrob Agents Chemother. 2017;61(4).

8. Fang J, Li H, Zhang M, et al. Efficacy of Ceftazidime-Avibactam Versus Polymyxin B and Risk Factors Affecting Clinical Outcomes in Patients With Carbapenem-Resistant Klebsiella pneumoniae Infections a Retrospective Study. Front Pharmacol. 2021;12:780940.

9. Chen J, Liang Q, Chen X, et al. Ceftazidime/Avibactam versus Polymyxin B in the Challenge of Carbapenem-Resistant Pseudomonas aeruginosa Infection. Infect Drug Resist. 2022;15:655-667.

10. Zheng G, Cai J, Zhang L, et al. Ceftazidime/Avibactam-Based Versus Polymyxin B-Based Therapeutic Regimens for the Treatment of Carbapenem-Resistant Klebsiella pneumoniae Infection in Critically Ill Patients: A Retrospective Cohort Study. Infect Dis Ther. 2022;11(5):1917-1934.

11. Chang K, Wang H, Zhao J, et al. Polymyxin B/Tigecycline Combination vs. Polymyxin B or Tigecycline Alone for the Treatment of Hospital-Acquired Pneumonia Caused by Carbapenem-Resistant Enterobacteriaceae or Carbapenem-Resistant Acinetobacter baumannii. Front Med (Lausanne). 2022;9:772372.

12. Akajagbor DS, Wilson SL, Shere-Wolfe KD, et al. Higher incidence of acute kidney injury with intravenous colistimethate sodium compared with polymyxin B in critically ill patients at a tertiary care medical center. Clin Infect Dis. 2013;57(9):1300-1303.

13. Aggarwal R, Dewan A. Comparison of nephrotoxicity of Colistin with Polymyxin B administered in currently recommended doses: a prospective study. Ann Clin Microbiol Antimicrob. 2018;17(1):15.

14. Satlin MJ, Kubin CJ, Blumenthal JS, et al. Comparative effectiveness of aminoglycosides, polymyxin B, and tigecycline for clearance of carbapenem-resistant Klebsiella pneumoniae from urine. Antimicrob Agents Chemother. 2011;55(12):5893-5899.

15. Quintanilha JCF, Duarte NDC, Lloret GR, et al. Colistin and polymyxin B for treatment of nosocomial infections in intensive care unit patients: pharmacoeconomic analysis. Int J Clin Pharm. 2019;41(1):74-80.

16. Wang J, Shah BK, Zhao J, et al. Comparative study of polymyxin B and colistin sulfate in the treatment of severe comorbid patients infected with CR-GNB. BMC Infect Dis. 2023;23(1):351.

17. Tang T, Li Y, Xu P, et al. Optimization of polymyxin B regimens for the treatment of carbapenem-resistant organism nosocomial pneumonia: a real-world prospective study. Crit Care. 2023;27(1):164.

18. Liu S, Wu Y, Qi S, et al. Polymyxin B therapy based on therapeutic drug monitoring in carbapenem-resistant organisms sepsis: the PMB-CROS randomized clinical trial. Crit Care. 2023;27(1):232.

19. Yang J, Liu S, Lu J, et al. An area under the concentration-time curve threshold as a predictor of efficacy and nephrotoxicity for individualizing polymyxin B dosing in patients with carbapenem-resistant gram-negative bacteria. Crit Care. 2022;26(1):320.

20. Junyan Qu, Jian Xu, Yanbin Liu, et al. Real-world effectiveness of ceftazidime/avibactam versus polymyxin B in treating patients with carbapenem-resistant Gram-negative bacterial infections. Int J Antimicrob Agents. 2023;62.

21. Wu J, Liu T, Yuan Y, et al. The evaluation of clinical efficacy and safety of polymyxin B in treatment of senile patients with hospital-acquired pneumonia caused by carbapenem-resistant bacteria. Medical Journal of Chinese People's Liberation Army. 2020.

22. Zha L, Zhang X, Cheng Y, et al. Intravenous Polymyxin B as Adjunctive Therapy to High-Dose Tigecycline for the Treatment of Nosocomial Pneumonia Due to Carbapenem-Resistant Acinetobacter baumannii and Klebsiella pneumoniae: A Propensity Score-Matched Cohort Study. Antibiotics (Basel). 2023;12(2).


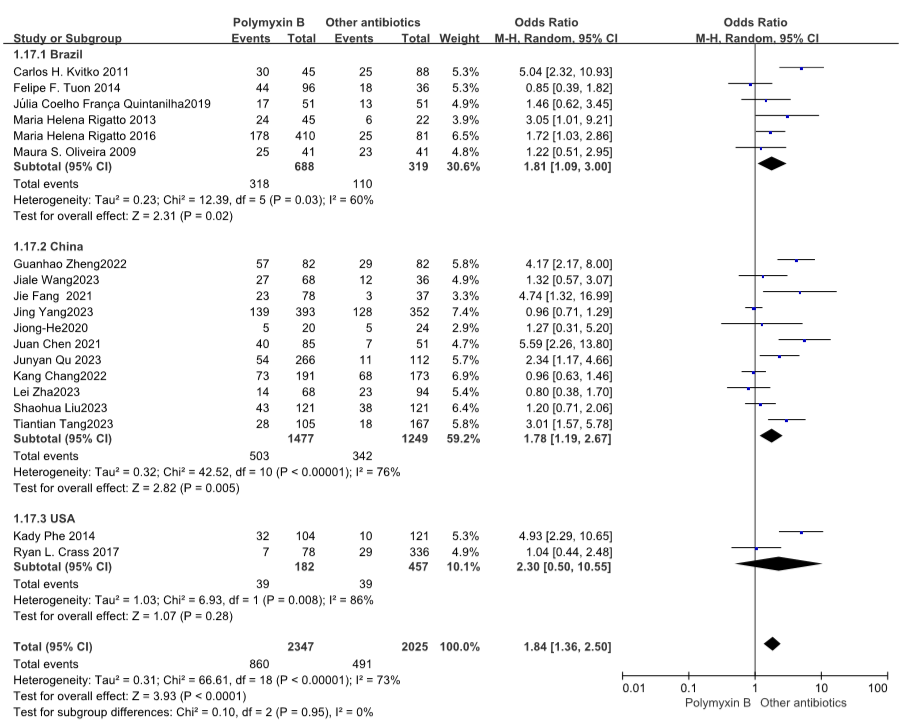


**Figure S1. The mortality was compared between PMB and other antibiotics (subgroup analysis based on different countries)**


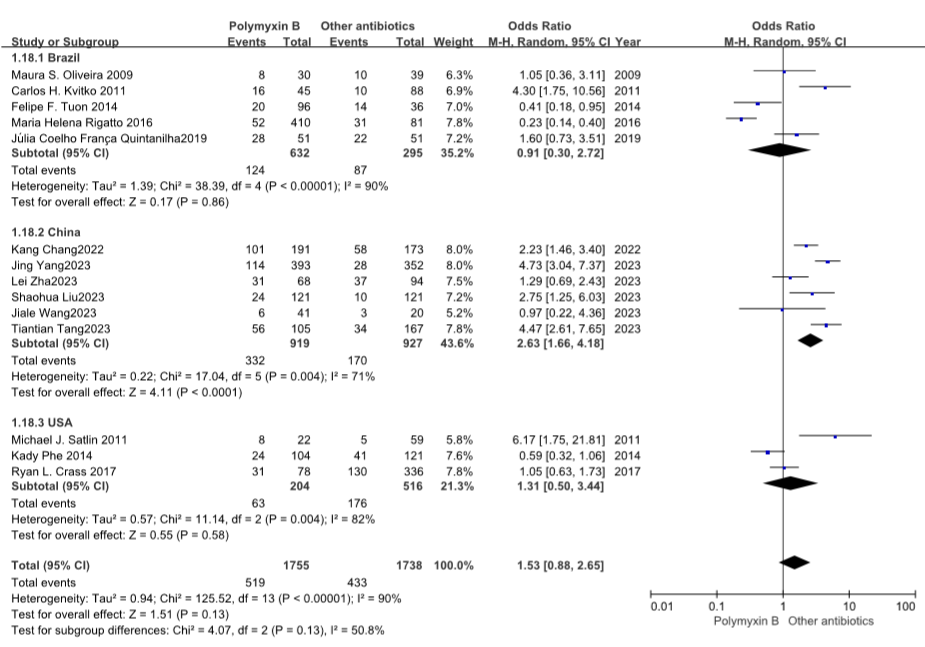


**Figure S2. The nephrotoxicity was compared between PMB and other antibiotics (subgroup analysis based on different countries)**
